# A Self‐Assembly Pro‐Coagulant Powder Capable of Rapid Gelling Transformation and Wet Adhesion for the Efficient Control of Non‐Compressible Hemorrhage

**DOI:** 10.1002/advs.202306289

**Published:** 2023-12-03

**Authors:** Xiong‐Xin Lei, Chen‐Yu Zou, Juan‐Juan Hu, Ming‐Hui Fan, Yan‐Lin Jiang, Ming Xiong, Chen Han, Xiu‐Zhen Zhang, Ya‐Xing Li, Long‐Mei Zhao, Rong Nie, Jesse Li‐Ling, Hui‐Qi Xie

**Affiliations:** ^1^ Department of Orthopedic Surgery and Orthopedic Research Institute Laboratory of Stem Cell and Tissue Engineering State Key Laboratory of Biotherapy West China Hospital Sichuan University Chengdu Sichuan 610041 P. R. China; ^2^ Frontier Medical Center Tianfu Jincheng Laboratory Chengdu Sichuan 610212 P. R. China; ^3^ Department of Otolaryngology – Head & Neck Surgery West China Hospital Sichuan University Chengdu Sichuan 610041 P. R. China; ^4^ Center of Medical Genetics West China Second University Hospital Sichuan University Chengdu Sichuan 610041 P. R. China; ^5^ Department of Orthopedic Surgery First People's Hospital of Foshan Foshan Guangdong 528000 P. R. China

**Keywords:** hemostatic powder, non‐compressible hemorrhage, pro‐coagulant bioactivity, self‐gelling, wet adhesion

## Abstract

Rapid and effective control of non‐compressible massive hemorrhage poses a great challenge in first‐aid and clinical settings. Herein, a biopolymer‐based powder is developed for the control of non‐compressible hemorrhage. The powder is designed to facilitate rapid hemostasis by its excellent hydrophilicity, great specific surface area, and adaptability to the shape of wound, enabling it to rapidly absorb fluid from the wound. Specifically, the powder can undergo sequential cross‐linking based on “click” chemistry and Schiff base reaction upon contact with the blood, leading to rapid self‐gelling. It also exhibits robust tissue adhesion through covalent/non‐covalent interactions with the tissues (adhesive strength: 89.57 ± 6.62 KPa, which is 3.75 times that of fibrin glue). Collectively, this material leverages the fortes of powder and hydrogel. Experiments with animal models for severe bleeding have shown that it can reduce the blood loss by 48.9%. Studies on the hemostatic mechanism also revealed that, apart from its physical sealing effect, the powder can enhance blood cell adhesion, capture fibrinogen, and synergistically induce the formation of fibrin networks. Taken together, this hemostatic powder has the advantages for convenient preparation, sprayable use, and reliable hemostatic effect, conferring it with a great potential for the control of non‐compressible hemorrhage.

## Graphical abstract

To control the non‐compressible hemorrhage, an efficient self‐assembly biocompatible powder is prepared. Owing to the ingenious materials’ design, it can rapidly gelling transform, realize wet adhesion and exert pro‐coagulant bioactivity, fully leveraging the fortes of powder‐ and hydrogel‐type. Moreover, the outstanding hemostatic performance is synthetically verified in rat, rabbit, and canine hemorrhage models, signifying it a promising hemostatic material.

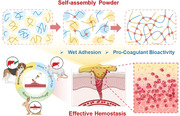

## 1 Introduction

Uncontrolled bleeding, which may occur during major surgery, war and natural disasters, has been a leading cause for death.^[^ ^1^ ^]^ And nearly 50% of such deaths could be prevented by effective hemostasis.^[^ ^2^ ^]^ A great effort has been made to develop hemostatic materials including blood products, coagulation factors, and antifibrinolytics, which have become the standard treatment for acute clinical bleeding. However, this is not enough for the effective management of non‐compressible hemorrhage from the body trunk and internal organs.^[^ ^3^ ^]^ To develop portable and efficient hemostatic materials has therefore become an urgent need, particularly for the management of non‐compressible intracavitary and junctional massive hemorrhages.

During the course of hemostasis, effective tissue adhesion and sealing are crucial, which can conduce to form a physical barrier at the bleeding site and prevent the blood loss.^[^ ^4^ ^]^ To address the challenges from the wet environment, several strategies for wet adhesion have been proposed, which included catechol chemistry, biomimetic microstructure and hydrophilic/hydrophobic design.^[^ ^5^ ^]^ Nevertheless, other numerous deficiencies, such as pH/temperature sensitivity, a complicated process, and poor adhesion strength, severely hinder their large‐scale production and clinical adoption.^[^ ^6^ ^]^ As a result, researchers have turned their attention toward the primary obstacles posed by the interfacial water, trying to solve them through a simple yet effective strategy.^[^ ^7^ ^]^ Zhao's team has designed a dry double‐sided adhesive tape for the removal of interfacial water from tissue surface, with an aim to achieve strong adhesion between the material and wet dynamic tissues.^[^ ^8^ ^]^ Similarly, to remove the fluid and adhesion with the tissue surface by absorbing massive blood through rational design could be a promising strategy for uncontrolled bleeding.

Powder‐type materials possess a great surface area and excellent ability for moisture absorption, making them a promising candidate for the development of hemostatic materials. These attributes may facilitate the swift absorption of blood by the powders and promote the clot formation, which can effectively halt the bleeding.^[^ ^9^ ^]^ Nevertheless, conventional hemostatic powders, capable of absorbing blood to promote hemostasis in bleeding wounds, are susceptible to dispersion and/or dissolution due to the blood flow. Their effectiveness for stopping the bleeding is therefore limited, as they may fail to establish a stable and durable physical barrier on the wound surface.^[^ ^10^ ^]^ Powder materials, which can transform to gel upon contact with the blood, a strategy known as self‐gelling, can attain the goals for fluid removal, tissue sealing, and hemostasis. Researches have demonstrate that, in a wet status, the powder can undergo powder‐gel conversion through physical cross‐linking facilitated by hydrogen bonding.^[^ ^11^ ^]^ However, the stability of intermolecular forces under the complicated conditions in the blood may still pose a challenge.^[^ ^5^, ^12^ ^]^

“Click” chemistry and the dynamic covalent bonds have been widely applied for material modification and drug design for their fast reaction rate, strong bonding strength, and reversible characteristics.^[^ ^13^ ^]^ Fang et al. have recently described a bi‐component powder which could self‐cross‐link into an adhesive hydrogel within 10 s. Such rapid powder‐gel transition performance was in part attributed to the formation of Schiff‐base linkages.^[^ ^14^ ^]^ Similarly, based on highly efficient cross‐linking reaction between the NHS esters and the primary amine groups, a gelable powder is developed and can halt the severe bleeding from pig visceral organs.^[^ ^15^ ^]^ Thus, “click” chemistry and the dynamic covalent bonds could offer a new strategy for attaining the goals of rapid gelling, sealing irregular wounds and resisting the blood pressure.

**Figure 1:**
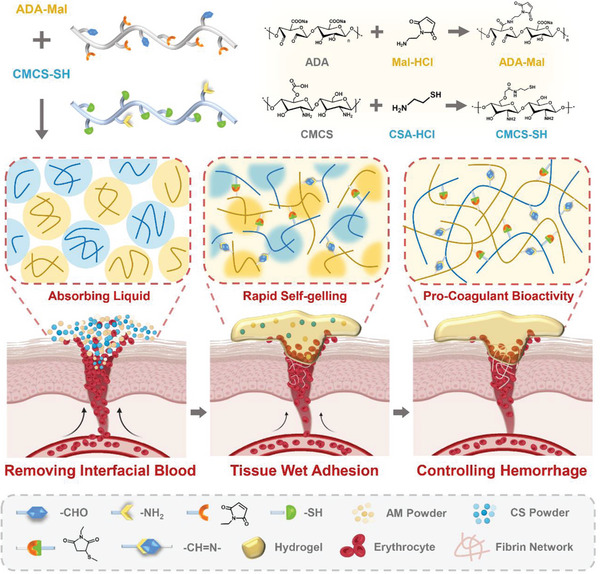
Schematic representation of the chemical synthesis process of ADA‐Mal and CMCS‐SH and the preparation of rapid self‐gelling powder for the control of non‐compressible massive hemorrhage.

Enlightened by the aforementioned perspectives, this study has aimed to develop an efficient assembly hemostatic powder comprised of hydrophilic biomacromolecules, namely sodium alginate (SA) and carboxymethyl chitosan (CMCS). The powder has shown rapid self‐gelling, wet adhesion, pro‐coagulation activity and sprayable use characteristics (**Figure** **1**). It can rapidly absorb the blood upon contact, followed by dissolving and exposing the active groups contained by the powder, and undergoing cross‐linking through sequential reactions involving thiol‐Michael addition and Schiff base reaction and self‐gelation within 10 s, effectively sealing the wound and attain the goal of hemostasis. The efficient removal of interfacial tissue fluid and mediation of material‐tissue interactions have enabled reliable tissue wet adhesion and sealing, and attained a significant hemostatic effect in non‐compressible severe hemorrhage models constructed with rat, rabbit, and beagle dogs. Through deep inquiry, in addition to wet tissue adhesion, the powder could also synergistically induce fibrin network formation through promoting the adhesion of blood cells and capturing fibrinogen. The unique design of this self‐gelling powder may also offer inspiration for the development of next‐generation hemostatic agents and functional dressings.

## 2 Results and Discussion

### 2.1 Preparation and Characterization of the Powders

To achieve the rapid formation of the hydrogels by click chemistry, the CMCS‐SH (CMCS grafted sulfhydryl group) and ADA‐Mal (SA grafted aldehyde and maleimide groups) were first synthesized. Specifically, the aldehyde groups were introduced at the C2 and C3 positions of the sugar carboxylic acid units in the SA by NaIO_4_ in an ethanol‐water solution. And the amidation catalyzed by EDC/NHS was carried out to introduce the sulfhydryl and maleimide groups into the CMCS and ADA, respectively.

**Figure 2:**
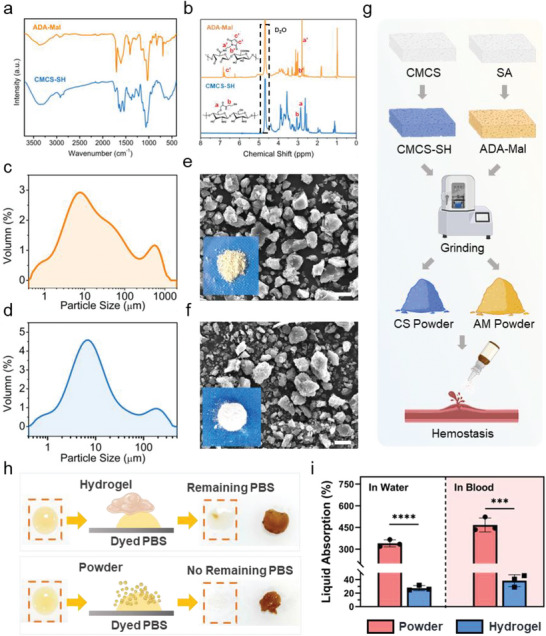
a) FTIR and b) ^1^H NMR spectra of the modified sodium alginate (ADA‐Mal) and modified carboxymethyl chitosan (CMCS‐SH). The distribution of particle size of c) ADA‐Mal and d) CMCS‐SH as detected by DLS. SEM and macroscopic images of e) ADA‐Mal and f) CMCS‐SH, Scale bar = 10 µm; g) Diagram for the powder preparation using a convenient liquid nitrogen‐assisted mechanical grinding method; h) Evaluation of PBS absorption for the A7C3 powder and A7C3 hydrogel; i) Liquid absorption (%) of the A7C3 powder and hydrogel in the PBS and blood (*n* = 3) (***P < 0.001, ****P < 0.0001).

The chemical structures of ADA‐Mal and CMCS‐SH were evaluated by proton nuclear magnetic resonance (^1^H NMR) and Fourier Transform Infrared (FTIR). As shown in Figure S1 (Supporting Information), the peak at 1734 cm^−1^ ascribed the stretching vibration of C═O in the aldehyde group may be seen in the FTIR spectrum of ADA. As determined by a hydroxylamine hydrochloride method, ^[^ ^16^ ^]^ the oxidation degree of the oxidative SA (ADA) was 33.3%. As shown in **Figure** **2**a, the absorption peaks at 1556 and 1648 cm^−1^ of the FTIR spectra of the ADA‐Mal were both generated by the amido bonds, which suggested that the amino group in maleimide hydrochloride has reacted with the carboxyl group in the ADA. And a strong absorption peak at 1700 cm^−1^ may correspond to the stretching vibration of C═O bonds in the maleimide groups.^[^ ^17^ ^]^ Similarly, the peaks attributed to the amide bonds (1613 and 1540 cm^−1^) may be seen in the spectrum of CMCS‐SH. As shown in Figure 2b, the appearance of proton peaks (δ = 2.88, 2.95, and 6.88 ppm) in the ADA‐Mal and the peaks (δ = 2.86 and 2.91 ppm) in the spectra of CMCS‐SH had ulteriorly confirmed the grafting of functional groups. Furthermore, the substitute ratio of the functional groups was determined by ^1^H NMR. The maleimide group substitute ratio of the ADA‐Mal, calculated as the ratio of integrated area of peaks (δ = 6.7–6.9 ppm) to that of the peaks at 3.4–4.4 ppm, was 22%. Analogously, with CMCS‐SH, the substitute ratio of sulfhydryl groups was 20% according to the ratio of integrated area of peaks (δ = 2.8–3.0 ppm) to that of the CMCS backbone's peaks at 3.3–4.2 ppm. Taken together, above results indicated that the synthesis of CMCS‐SH and ADA‐Mal was successful.

The ADA‐Mal and CMCS‐SH powders were prepared using a convenient liquid nitrogen‐assisted mechanical grinding method which may facilitate large‐scale production and product conversion (Figure 2g). As shown in Figure 2c,d, the size of ground powders as assessed by dynamic light scattering (DLS) has shown a wide distribution. The median size (D50, the corresponding particle size when the cumulative particle size distribution percentage of the sample reaches 50%) of the ADA‐Mal and CMCS‐SH powders were 7.11 and 7.39 µm, respectively. The SEM images (Figure 2e,f) revealed that the shape of the powders was mainly irregular polyhedron, and the powder size is around 10 µm, in accordance with the DLS results.

The A7C3, A5C5 and A3C7 were prepared by mixing the ADA‐Mal and CMCS‐SH powders at the mass ratios of 7 : 3, 5 : 5 and 3 : 7, respectively. The powder‐type hemostatic materials have a time‐proven and wide application owing to their merits of adapting to the irregular topography of the wound and absorbing a large amount of blood.^[^ ^18^ ^]^ Hence, the water absorption capacity was primarily evaluated between the different forms (hydrogel and powder) of the same material. As shown in Figure 2h, 300 µL of dyed PBS was absorbed by the A7C3 hydrogel and powder of the same weight. Unlike the residual PBS in the hydrogel group, the powder‐type materials could absorb the PBS within 3 min. The liquid absorption behavior of the A7C3 hydrogel and powder in the PBS and blood was evaluated. The PBS/blood absorption curves in Figure S2 (Supporting Information) showed a significant difference between the powder and hydrogel. After 15 min, the absorption ratio of the PBS and blood in Figure 2i has reached 339.89 ± 24.14% and 467.35 ± 48.14% for the A7C3 powder, and 27.24 ± 3.75% and 38.29 ± 8.71% for the A7C3 hydrogel, respectively. The powder was comprised of natural biopolymers and abundant hydroxyl, amino, carboxyl and other hydrophilic groups, and has a large surface area compared with the hydrogel, therefore possessed excellent liquid absorption capacity and is suitable for treating massive hemorrhage.

### 2.2 Rapid Gelling Transformation of the Powders

**Figure 3:**
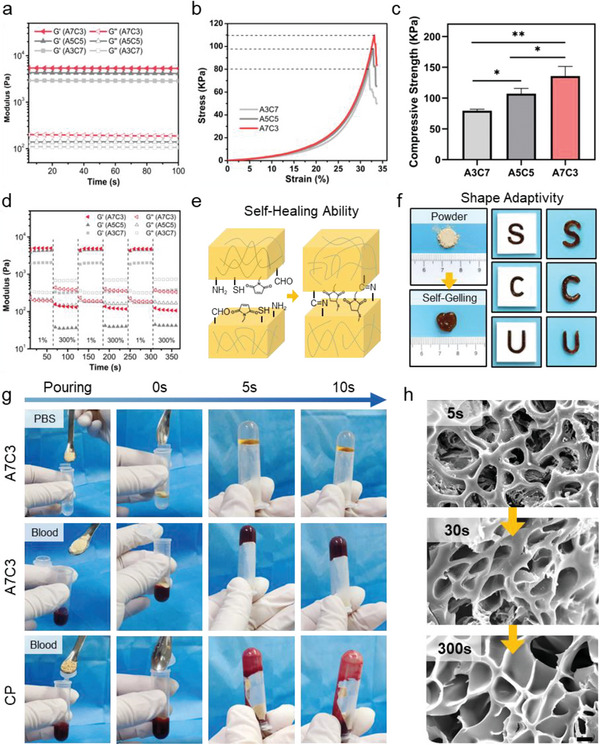
a) Rheological curves of the A3C7, A5C5, and A7C3 under time sweep mode, and schematic diagram of sequential cross‐linking formation; b) Stress‐strain curves in compression test and c) compressive strength (KPa) of the A3C7, A5C5, and A7C3 (*n* = 3); d) Rheological curves of the three groups under alternate step strain sweep mode, and e) scheme of self‐healing ability; f) Shape adaptivity of the A7C3 as evaluated by the “S”, “C”, “U”‐shaped models; g) Inverted tube method to illustrate the self‐gelling effect in PBS and blood; h) SEM images of the A7C3 during the hydrogel‐transformed process, Scale bar = 10 µm (*P < 0.05, **P < 0.01).

After absorbing the blood, the ability of the powder to transform into a stable hydrogel state quickly, on the one hand, is conducive to the formation of a physical barrier for blood pressure and further promote the hemostasis. On the other hand, it can prevent the powder from entering the blood circulation, which may cause additional safety risks.^[^ ^19^ ^]^ The rapid self‐gelling capability of the A3C7, A5C5, and A7C3 powders was evaluated by rotational rheometer. Under the time sweep mode shown in Figure **3**a, hydrogels after powder self‐gelling have exhibited the typical viscoelasticity, where the storage modulus (G’) was greater than the loss modulus (G’’). The G’ of the A7C3 powder was 5377 Pa, which was approximately 1.86 and 1.27 times those of the A3C7 and A5C5, suggesting that a denser hydrogel network has formed in the A7C3 powder. An uniaxial compression experiment was carried out to evaluate the static mechanical properties of the A3C7, A5C5, and A7C3 powders. As shown in Figure 3b,c, the compressive strength of the A3C7, A5C5, and A7C3 was 79.50 ± 2.41, 107.1 ± 8.72, and 135.7 ± 15.62 KPa, respectively, which suggested that the powder can form a solid three‐dimensional network structure after absorbing the water, which is conductive to the resistance of external stress. Furthermore, the swelling and degradation tests could reflect the stability of the material, and the swelling and degradation curves of the A3C7, A5C5, and A7C3 were shown in Figure S3 (Supporting Information). Among these, the A7C3 showed the highest remaining mass (52.90 ± 5.55% on day 21) and lowest swelling rate (43.16 ± 10.86% at 48 h), proving that it has the most stable hydrogel structure, which is conducive to the formation of a stable physical barrier on the wound surface and ensured effective hemostasis. The A7C3 powder showed the superior mechanical property and appropriate stability, indicating that the gel with 7 : 3 composition ratio of ADA‐Mal and CMCS‐SH exhibited superior cross‐link strength and cohesion. Since, good cohesion is the basis of tissue adhesion, to enhance the cohesion is also an effective strategy to improve the tissue adhesion strength.^[^ ^20^ ^]^

We have further analyzed the formation mechanism of the hydrogel by X‐ray photoelectron spectroscopy (XPS). As shown in Figure S4 (Supporting Information), the changes in chemical bonds were emphasized in the C 1s, N 1s, and S 2p spectrum. The peaks of C─S bonds could be seen in C 1s (286.4 eV) and S 2p (164.6 eV), which suggested the sulfhydryl‐Michael addition reaction, and the peaks located in 285.7 and 400.5 eV of C 1s and N 1s were respectively attributed to the C═N bonds forming through the Schiff base reaction. In this study, the click chemistry was elaborately applied to achieve rapid self‐gelling. Among them, the formation of thioether bonds is rapidly mediated by the sulfhydryl groups in CMCS‐SH with nucleophilic groups (e.g., ‐Mal and aldehyde groups in ADA‐Mal) in a sulfhydryl‐Michael addition reaction to generate the initial cross‐linked network of the hydrogel. Simultaneously, according to Schiff base reaction, the aldehyde group in ADA‐Mal forms an imide bond with the amine group in CMCS‐SH, forming the dual‐cross‐linking network in the hydrogel. This sequential cross‐linking is due to the fact that sulfhydryl groups are much more nucleophilic than amino groups, which means that they react more readily with electrophilic groups, and the sulfhydryl‐Michael addition reaction mediated by them is more rapid.^[^ ^17^ ^]^ Such formation of dual‐cross‐linking network in hydrogel is conducive to the efficient gelling transformation followed by fully dissolution and stabilization of the material on the bleeding wound.

To apply the hydrogels for conditions with massive bleeding and tackle the potential damages caused by blood pressure, tearing and external force, we have further explored the self‐healing behavior of the A3C7, A5C5 and A7C3. ^[^ ^21^ ^]^ As shown in Figure 3d, the hydrogel network has been maintained (G’>G’’) under a low strain (1%) and destroyed (G’<G’’) under an excessive strain (300%), and the integrity of the hydrogel can be restored repeatedly during the alternation of large and small strains. This self‐healing behavior may be attributed to the click‐cross‐linking mechanism (Figure 3e). Based on our design, the imide and thioether bonds formed by the Schiff base and sulfhydryl‐Michael addition reaction are reversible, which could recover in a short time even if being damaged under severe conditions. Furthermore, to test the shape adaptivity, “S”, “C” and “U”‐shaped models were applied to simulate irregular and deep wound surface, which is more similar to the actual situation. As shown in Figure 3f, after removing the models with different forms, all samples have remained the intact original shape and undamaged, mainly due to the rapid self‐gelling and self‐healing abilities of the powders.

To illustrate the significance of introducing both the sulfhydryl‐Michael addition reaction and Schiff base reaction, SA‐Mal was prepared initially. The ‐Mal groups were grafted into the sodium alginate, not into the ADA. As shown in Figure S5a,b (Supporting Information), the ADA and CS did not react quickly, at least for 5 min, which has hampered the rapid gelation through the Schiff base reaction alone and diminished its value for emergency hemostasis. As for the SA‐Mal+CS hydrogel, the gelling time was 14.67 ± 1.53 s, which suggested that the sulfhydryl‐Michael addition reaction has occurred faster than the Schiff base reaction. When the two reactions were introduced to the hydrogel, the gelling time has decreased to 5.00 ± 1.00 s, which has highlighted the importance of both reactions. As shown in Figure S5c,d (Supporting Information), the adhesive strength of the ADA‐Mal + CS and SA‐Mal + CS hydrogels was 89.57 ± 6.62 KPa and 52.57 ± 15.83 KPa, which were 11.2 and 6.6 times that of the ADA+CS hydrogel, respectively. For the ADA + CS hydrogel, the partial aldehyde groups in the ADA could have contributed to the adhesion, but owing to the single hydrogel network, the cohesion and adhesion effect was poorer. These indicated that the dual‐cross‐linking network formed through the sulfhydryl‐Michael addition reaction and Schiff base reaction has played an important role in the rapid gelation and mechanical properties enhancement of the hydrogels.

Comprehensively, taking A7C3 as an example, the self‐gelling effect in PBS and blood were also manifested via inverted tube method, and the commercial powder (CP, Yunnan BaiYao), a widely used and effective hemostatic powder comprising of traditional Chinese medicine, was used as the control.^[^ ^22^ ^]^ As shown in Figure 3g and Movie S1 (Supporting Information), the A7C3 and CP with equal mass have promptly absorbed much liquid when they were put into a tube with PBS or blood. After inverting the tube for 5 s, the A7C3 has transformed the hydrogel and formed a intact barrier for the flow of liquid. Nevertheless, the blood in CP group has outflown. The absence of self‐gelling capacity of CP has resulted in easy disperse by blood flow, even though it can absorb liquid, which has seriously hampered its hemostatic effect. The structure change of A7C3 during the self‐gelling process under the SEM was shown in Figure 3h. In 5 s, the typical porous structure of the hydrogel has initially formed, and the uniform structure with connected pores has appeared in 30 s. For patients with penetrating trauma, there are only “platinum 5 min” in which effective treatment must be administered or the consequences will be fatal. Thus, the self‐gelling powder buys a great deal of time to save death.^[^ ^23^ ^]^

### 2.3 Tissue Wet Adhesion and Sealing of the Powders

**Figure 4:**
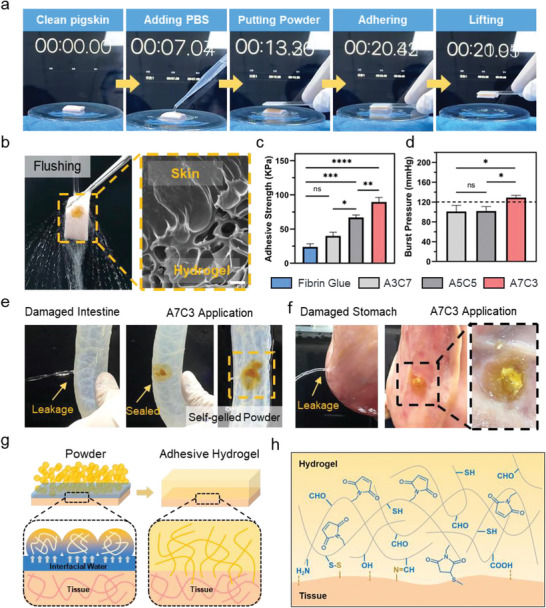
a) Adhesion of the A7C3 fixed on a glass onto a wet pigskin in a short time; b) Water scouring the pigskin adhered to the A7C3 and SEM images of the adhesive interface, Scale bar = 30 µm; c) The adhesive strength (KPa) and d) burst pressure (mmHg) of the fibrin glue, A3C7, A5C5, and A7C3 (*n* = 3) (the dashed line marks the normal human blood pressure); Photographs of A7C3 sealed damage of porcine e) small intestine and f) stomach; Schematic illustration of g) the removing interfacial water and self‐gelling of A7C3 and h) the wet tissue adhesion of the A7C3 based on multiple covalent and non‐covalent interactions (*P < 0.05, **P < 0.01, ***P < 0.001, ****P < 0.0001, ns: no significant difference).

The importance of tissue wet adhesion for the treatment of hemorrhage has been gradually recognized.^[^ ^24^ ^]^ Primarily, the wet bio‐adhesion effect was evaluated using pigskin revealed in **Figure** **4**a. To create a wet environment, the PBS was added onto the pigskin, and interestingly, it was lifted by a glass coated by the A7C3 within 10 s. Moreover, following immersing the two pigskins into PBS, they were covered by the powder and then put them together. As shown in Figure S6 and Movie S2 (Supporting Information), the two pieces of pigskin have firmly adhered together and can be lifted with a tweezer, which demonstrated excellent wet tissue adhesion. Furthermore, consider that when used in severe bleeding situations, hemostatic powders are subject to the impact of fast‐flowing blood. The resistance to blood scour of the powders was assessed in Figure 4b and Movie S3 (Supporting Information). The powder following the self‐gelling could tightly adhered on the tissue even if the violent current washed. And the tight adhesion at the hydrogel‐tissue interface was observed by SEM, showing a close connection microscopically. In order to quantitatively evaluate the tissue adhesion, the lap shear test was conducted according to the modified ASTM F2255‐05. In Figure 4c and Figure S7 (Supporting Information), the adhesion strength of A7C3 was as high as 89.57 ± 6.62 KPa, which was 3.75 times that of commercially made fibrin glue (*P*<0.0001). Among the three experimental groups, the A7C3 has attained the optimal adhesion strength, and the reason for this is twofold. Above all, the increased proportion of ADA‐Mal component can provide more aldehyde and maleimide groups, which can form more firm covalent bond cross‐linking with tissue. Second, based on the results of rheological and uniaxial compression experiments, the A7C3 has possessed the best mechanical property. Taken together, our self‐gelling powder has wet tissue adhesion and can adhere firmly to various tissue surfaces. Furthermore, the gelling time and adhesive strength of the A7C3 powder was compared with that of recently developed self‐gelling powders. As shown in Figure S8 (Supporting Information), the A7C3 powder has attained gelation transformation within 5 s, and its adhesive strength has reached 89.57 ± 6.62 KPa. To our knowledge, among all self‐gelling powders, the A7C3 powder has been the only one comprised entirely of natural polymers with an adhesive strength exceeding 60 KPa. These advantages are critical for rapid wound closure and effective hemostasis.

A bursting test was used to determine the tissue sealing ability. After stabilized for 30 min, use a syringe pump to increase the burst pressure. As shown in Figure 4d, the burst pressure of the A7C3 was 129.00 ± 4.50 mmHg, which was nearly 1.27 times than that of the A3C7 and A5C5, suggesting a greater resistance to blood scour. Besides, the burst pressure of the A7C3 was even higher than the normal systolic blood pressure of humans (120 mmHg), which may be attributed to the optimal cohesion and adhesion strength of the A7C3 powder.^[^ ^25^ ^]^ Accordingly, the prominent burst pressure confirms that the A7C3 powder may be particularly beneficial for the effective control of massive bleeding. The tissue sealing of the powder was further evaluated using perforating pig small intestine and stomach. In Figure 4e,f, the leakage was stopped after the hole was plugged with the powder, although the water was added into the tissue again. By combining the above factors, the A7C3 powder was selected for further experiments. The excellent wet tissue adhesion of this powder‐hydrogel transition form could be explained with the following factors:

As shown in Figure 4g, first of all, as a prominent advantage, the powder could well adapt to the complex morphology of tissues. Second, due to the excellent liquid absorption capacity, the interfacial liquid such as blood and body fluid on the wound is quickly absorbed, which could provide an environment suitable for tissue adhesion. In addition, after absorbing the water, the self‐gelling process could occur within 5 s, which could form a stable physical barrier on the wound surface. Importantly, the cohesion strength of the barrier has increased gradually due to the sequential formation of dual‐cross‐linking network. At the initial stage, the hydrogel network formed mainly by the sulfhydryl‐Michael reaction is conducive to entangling and sealing of the tissue. The formation of dual‐network hydrogel will strengthen the cohesion over time, which is conducive to stronger adhesion effect. Finally, hydrogels may form strong wet adhesion to tissues through two aspects. On the one hand, after liquid absorption, the interference of interfacial water is avoided, and the dissolved polymer chain in the materials can form better physical entanglement with tissues. On the other hand, a variety of reactive groups such as aldehyde, sulfhydryl, carboxyl and maleimide groups in the material could combine firmly with the tissue through covalent and non‐covalent interactions such as hydrogen bonding (Figure 4h).

Taken together, the excellent tissue wet adhesion and sealing effect of our powder may be attributed to the synergy of its water absorption capacity, rapid self‐gelation, sequential cross‐linking and multiple bio‐adhesion design.

### 2.4 In Vitro and In Vivo Biocompatibility of the Powders

**Figure 5:**
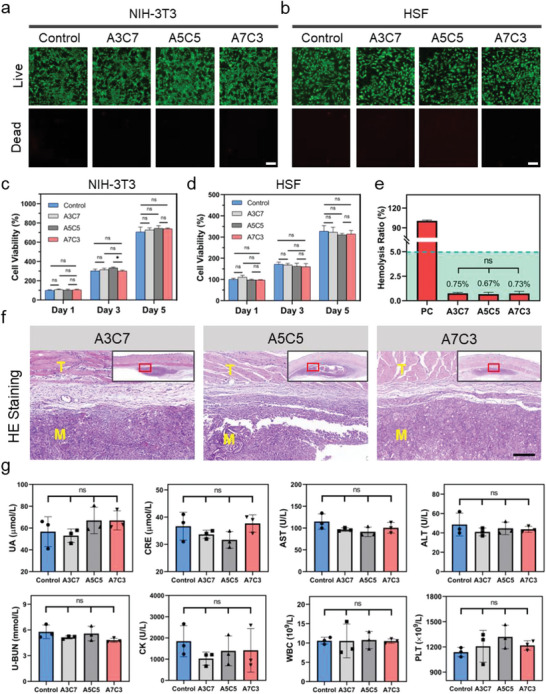
Live/Dead staining fluorescence images of a) NIH‐3T3 cells and b) HSF cells after incubation with the abstract of A3C7, A5C5, and A7C3 for 3 days, Scale bar = 200 µm; The effects of A3C7, A5C5, and A7C3 on the proliferation of the c) NIH‐3T3 cells and d) HSF cells (*n* = 3); e) Hemolysis ratio of the hydrogels (PC: 0.1% Triton X‐100) (*n* = 3); f) H&E staining after 7 days’ implantation of various materials, Scale bar = 200 µm (T: tissue, M: material); g) Blood routine and biochemistry examinations of the rats 7 days after the subcutaneous implantation (*n* = 3) (*P < 0.05, ns: no significant difference).

Evaluation of in vitro and in vivo biocompatibility, as the key characteristics of the materials, is imperative in the research of biomaterials.^[^ ^26^ ^]^ For assessing the cytocompatibility, CCK‐8 assay and Live/Dead staining were used to evaluate cell proliferation and cell growth activity, respectively. As shown in **Figure** **5**a,b, cultured in the extract of A7C3, A5C5, and A3C7 for 3 days, the NIH‐3T3 and HSF, the fibroblasts from mouse and human have respectively exhibited a vibrant status with typical long‐spindle shape stained by Calcein‐AM. Correspondingly, the dead cells stained by PI was almost invisible. The results of CCK‐8 assay in Figure 5c,d showed that the cells treated by the extract had similar proliferation capacity as the normal cells (the control group). However, there was no significant statistical difference among A7C3, A5C5, and A3C7 (*P*>0.5). The ideal hemostatic material should not cause hemolysis when contact with the bleeding wound. By the hemolytic test in Figure 5e, the hemolysis ratio, reflecting the extent of erythrocyte rupture, was less than 5% in all groups (*P*>0.5). Above results have indicated that the powders possessed satisfactory in vitro biocompatibility and could support the normal physiological state of cells.

To further assess their histocompatibility, the sterile prefabricated A7C3, A5C5, and A3C7 hydrogels were subcutaneously implanted in the rat notum. As shown by the H&E staining in Figure 5f, one week after the implantation, the implant was infiltrated by many inflammatory cells, and no obvious fibrous cyst was formed, which suggested moderate material‐host interaction. With the degradation of materials, the degradation product will enter the blood circulation for metabolism. Furthermore, a series of systemic toxicity evaluation including H&E staining of major organs, blood biochemistry and blood routine was carried out. In Figure S9 (Supporting Information), the degradation product of the A7C3, A5C5, and A3C7 implanted for 1 week has caused no pathological change in the heart, liver, spleen, lung and kidney. As shown in Figure 5g, blood biochemistry has indicated that the kidney function (e.g., UA, CRE, U‐BUN), liver function (e.g., AST and ALT) and myocardial injury (e.g., CK) were not different from those of healthy rats (*P*>0.05). Moreover, no significant increase in the white blood cells (WBC) and platelets (PLT) was noted, suggesting that there has been no change in the level of inflammation and clotting function. Furthermore, the biodegradation of the A7C3 powder has been investigated through subcutaneous implantation in rats. As shown in Figure S10 (Supporting Information), by both gross observation and ultrasonography, the size of the material has gradually decreased from day 7 to day 21. And the thickness of the implant was 0.37 ± 0.04 cm on day 21 and 49.1% on day 7, which indicated the biodegradability of the A7C3 powder. Taken together, through various in vitro and in vivo evaluation, the A7C3, A5C5, and A3C7 hydrogels have all shown sufficient biocompatibility and biodegradation for the subsequent in vivo experiments.

### 2.5 Non‐Compressible Hemorrhage Control of the Powders

**Figure 6:**
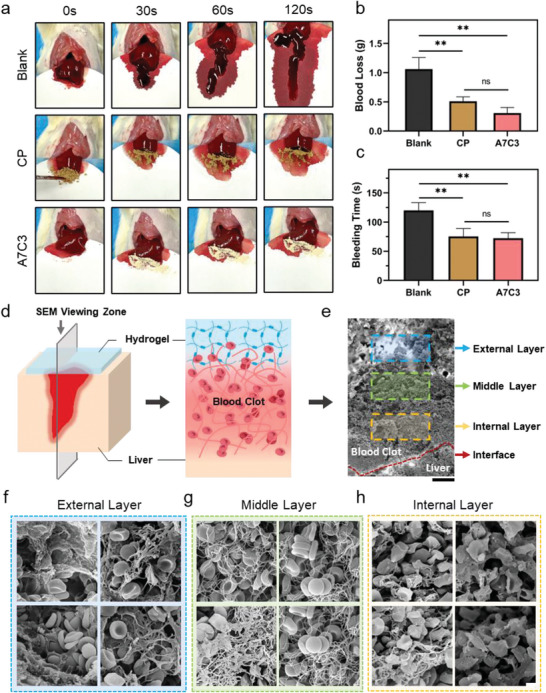
Photograph of a) hemostasis process, b) blood loss (g) and c) bleeding time (s) treated by CP and A7C3 in the rat bleeding liver model (*n* = 3). d) Schematic diagram of the viewing zone and e) SEM image of the liver bleeding site, Scale bar = 100 µm; SEM images of corresponding f) external, g) middle, and h) internal layers, Scale bar = 2 µm (**P < 0.01, ns: no significant difference).

The hemostatic effect of the powder was primarily evaluated with the rat liver resection model, with the CP as the positive control. As shown in **Figure** **6**a, the blood has flown out quickly when the liver was resected in the blank group. However, once the hemostatic agents (CP and A7C3) were applied, severe hemorrhage was quickly stopped, which suggested effective hemostasis. The blood loss and bleeding time were quantified in Figure 6b,c. In the A7C3 group, the blood loss was 0.31 ± 0.10 g, which was 70.48% lower than the blank group (*P*<0.01). Compared with positive control, the blood loss in the A7C3 group was also reduced, albeit with no significant difference (*P*>0.05). As for the bleeding time, the application of A7C3 has reduced it by 39.73% from 120.00 ± 13.23 s to 72.33 ± 9.29 s (*P*<0.01).

The bleeding site after the application of the A7C3 was also observed by SEM to analyze the hemostasis process. Based on the typical structures, the viewing zone was divided into external, middle and internal layers, as shown in Figure 6d,e. The three layers showed no clear boundaries in between, but have intertwined with each other to form a whole mass. In the external layer (Figure 6f), many erythrocytes were wrapped in the powder‐transformed hydrogel network. A large number of fibrin networks have interwoven with the hydrogel network to form a tight physical barrier. And the hydrogel layer has tightly adhered to the lower layer, suggesting a great pro‐coagulant activity and tissue adhesion for the A7C3. In the middle layer (Figure 6g), the overall structure was mainly tangled fibrin network, and the erythrocytes have aggregated and possessed a typical double‐concave disk shape, which was wrapped in the network. In the interior of the bleeding site (Figure 6h), the morphology of erythrocytes was changed greatly with polyhedral, and irregular erythrocytes were found mainly in the interior and rarely in the periphery, which was consistent with previous reports.^[^ ^27^ ^]^ Further analysis suggested that this may be due to the contractile stress produced by the pulling and bending fibrin fibers of the platelets. The trapped erythrocytes were forced to move closer to each other and deform. This change may be beneficial to create a impermeable barrier between the material and the bleeding tissue, which is important for the hemostasis and wound healing.

Notably, even though the A7C3 had less blood loss and bleeding time, no statistical difference was found between it and the CP group (*P*>0.05). A plausible explanation may be that the bleeding in rats was relatively mild, and the CP could effectively treat the bleeding in the rats. Therefore, it is necessary to further evaluate the hemostatic effect with large animal models for the non‐compressible hemorrhage.

**Figure 7:**
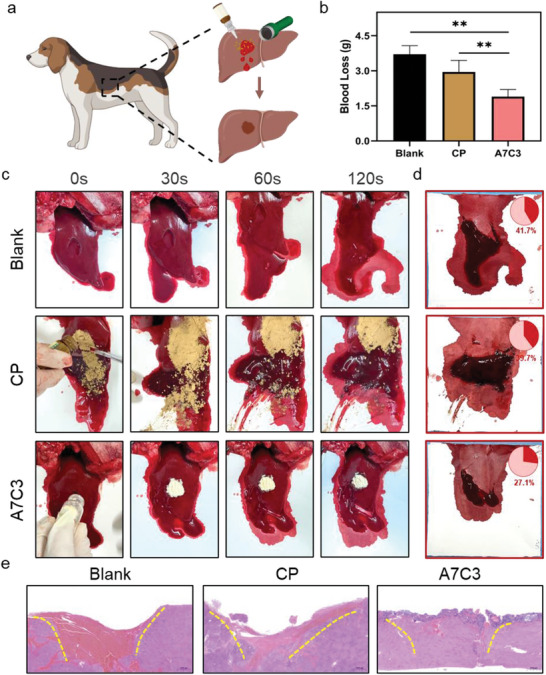
a) Schematic diagram of the canine liver defect non‐compressible hemorrhage model and the hemostat application; b) Blood loss and c) photograph of the hemostasis process in the blank, CP, and A7C3 groups (*n* = 3); d) Photograph of blood loss and the ratio of corresponding area with blood on the filter paper after hemostasis; e) H&E staining of liver after 1 week of hemostasis in different groups (**P < 0.01).

A rabbit model for the liver defect and central auricular artery bleeding was constructed for a proof‐of‐concept study. As shown in Figure S11a (Supporting Information), after creating a deep defect (1 cm) in the liver, the CP and A7C3 with the same mass (300 mg) were quickly added onto the bleeding wound. Compared with CP, bleeding in the A7C3 group was quickly controlled. As shown in Figure S11b (Supporting Information), the CP was washed away by the blood, which had hindered the hemostasis, and was not seen in the A7C3 group. This is because it could form an interconnected whole and lacks the ability to form a physical barrier, causing the failure of the control of deep defect bleeding. Moreover, the perforation caused by the syringe was used to construct the arterial bleeding model. Due to the high blood pressure, the bleeding in damaged arteries was violent even with the aid of weights (100 g). Depicted in Figure S11c (Supporting Information), the CP was scattered by the flowing blood and could not effectively stop the bleeding from the arteries. On the contrary, due to excellent liquid absorption, rapid self‐gelling and wet bio‐adhesion abilities, the A7C3 has retained its structure even in the face of such severe bleeding and fulfilled the task of hemostasis, and greatly reduced the amount of bleeding. Moreover, as shown in **Figure** **7**a, a non‐compressible massive hemorrhage model was created in the canine liver using rotary skin biopsy trephine. As shown in Figure 7b, the blood loss in the A7C3 group (1.89 ± 0.31 g) was only 51.11% and 64.21% that of the blank control and CP group (3.70 ± 0.37 g and 2.95 ± 0.49 g, respectively; *P*<0.01), which has suggested outstanding hemostasis. Apparently, as shown in Figure 7c, the amount of liver bleeding in the canine model was much greater than that in rats and rabbits. And the bleeding process after different treatment has indicated that, similar to the liver bleeding in rabbits, the A7C3 has rapidly self‐gelled following blood absorption and firmly adhered to the tissue. By contrast, the CP has failed to adhere to the bleeding site. After the hemostasis by A7C3, the amount of blood on the weighing paper has also decreased from 41.7% to 27.1% (Figure 7d). The adhesion of the material on the wound was further analyzed by H&E staining. As shown in Figure 7e, in the A7C3 group, a continuous barrier layer has formed on the defected liver surface, indicating excellent self‐gelation and tissue adhesion, and these were barely seen with the CP. It is worth mentioning that the liver defects have remained in the blank and PC groups, while the liver treated by the A7C3 has regenerated rapidly, indicating that it has a potential for liver repair. Through the verification with various bleeding models, we have confirmed that the A7C3 can quickly absorb flowing blood, create a stable physical barrier to seal the wound surface, and achieve a superior hemostasis and repairment effect for non‐compressible massive bleeding compared with the CP.

### 2.6 Pro‐Coagulant Activity and Hemostatic Mechanism of the Powders

**Figure 8:**
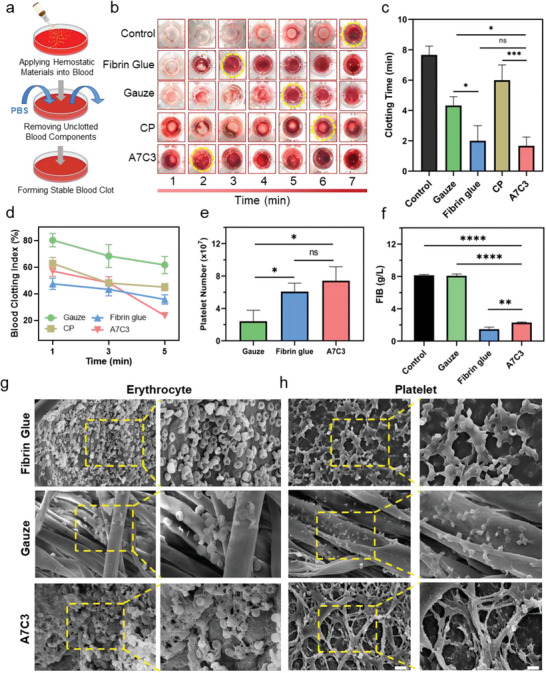
a) Schematic illustrations for the induction of blood clotting by adding hemostats into anticoagulant whole blood; b) Photographs of blood clotting following the application of fibrin glue, gauze, CP, and A7C3; c) The clotting time (min) of various materials (*n* = 3); d) The BCI (%) of the materials at various time points (1, 3, 5 min) (*n* = 3); e) The number of platelets adhered to the surface of gauze, fibrin glue and A7C3 (*n* = 3); f) The FIB (g/L) in plasma treated by gauze, fibrin glue and A7C3 (*n* = 3); SEM images of g) erythrocytes and h) platelets adhered to the fibrin glue, gauze and A7C3, Scale bars = 10 µm and 5 µm at low and high magnification, respectively (*P < 0.05, **P < 0.01, ***P < 0.001, ****P < 0.0001, ns: no significant difference).

The excellent hemostatic effect of the A7C3 was verified by bleeding models in rat, rabbit and canine. Compared with commercial hemostats including CP, gauze and fibrin glue, the A7C3 hydrogel has caused less blood loss and bleeding time, suggesting it a promising hemostatic material. Subsequently, the pro‐coagulant activity and hemostatic mechanism of the A7C3 were investigated. As shown in **Figure** **8**a, the hemostatic material was added to the re‐activated anticoagulant whole blood and the time required for the formation of stable thrombus was recorded. Remarkably, as shown in Figure 8b,c, the A7C3 has formed a blood clot in just 1.67 ± 0.58 min after contacting with the blood. By contrast, the clotting time for the control, fibrin glue, gauze and CP groups were 7.67 ± 0.58 min, 2.00 ± 1.00 min, 4.33 ± 0.58 min and 6.00 ± 1.00 min, respectively. The pro‐coagulant ability was further reflected by blood clotting index (BCI). The lower the BCI, the stronger the clotting tendency.^[^ ^28^ ^]^ As shown in Figure 8d, the BCI of gauze, CP, fibrin glue and A7C3 was time‐dependent and showed a decreasing trend with the extension of time from 1 to 3 min. On the whole, fibrin glue and A7C3 showed sound coagulation‐promoting activity, with the BCI at 5 min being 35.79 ± 3.45% and 23.56 ± 1.96%, respectively, which were in keeping with their outstanding in vivo hemostatic effect.

The blood clotting is a spontaneous and complex process which involves a variety of cells and substances. As a forerunner and component of the coagulation cascade reaction, the platelets play a crucial role in the whole system.^[^ ^24^, ^29^ ^]^ In the present study, to avoid the concentration effect caused by liquid absorption and assess the influence of the material itself on the coagulation, the hydrogel‐type material was pre‐fabricated. As shown in Figure 8e, the platelets adhered to the A7C3 have reached 7.42 × 10^7^ ± 1.73 × 10^7^, which was remarkably greater than that on the gauze surface (2.42 × 10^7^ ± 1.36 × 10^7^) (*P* < 0.05). Fibrin glue has been widely recognized as a hemostatic material for the promotion of platelet adhesion,^[^ ^30^ ^]^ and the number of platelets on its surface was 6.08 × 10^7^ ± 1.03 × 10^7^, which showed no significant difference from those on the A7C3. Moreover, different clotting pathways involved in the system may eventually lead to conversion of soluble fibrinogen (FIB) to insoluble fibrin, i.e., activation of Factor I.^[^ ^30^ ^]^ Hence, the effect of the A7C3 on another key substance, FIB, was evaluated. The content of FIB in plasma after various treatments was analyzed with an automatic coagulometer. As shown in Figure 8f, after being treated with the A7C3, the residual FIB in the plasma was 2.29 ± 0.07 g L^−1^, which was only 28% that of the control group. In other words, nearly 72% of the FIB in the original plasma has been adsorbed on the A7C3 surface, which may greatly increase the rate of fibrin network formation. The SEM has been used to evaluate the blood cells and fibrin networks formation on the fibrin glue, gauze and A7C3 (Figure 8g,h). Notably, significantly more erythrocytes and platelets have adhered to the A7C3 and fibrin glue, while a small number had appeared on the gauze. In the A7C3 group, the erythrocytes were covered with fibrin networks, a phenomenon barely found in the other two groups. Similarly, after the treatment with the fibrin glue and A7C3, the platelets were activated and involved in the construction of fibrin networks, and the fibrin in the A7C3 group was tighter and more complete than that in the fibrin glue group. Above results have suggested that the A7C3 had a pro‐coagulant activity. The mechanisms by which A7C3 can promote blood cells adhesion and formation of fibrin networks may include: First, CMCS has a positive charge and can attract negatively charged platelets through electrostatic interaction. Second, the sulfhydryl and aldehyde groups could activate platelets and bind with proteins through imine bonds.^[^ ^31^ ^]^ In addition, the A7C3 has an innovative use, namely rapid blood absorbing and self‐gelling, which can effectively concentrate hemostasis‐related substances and seal the bleeding wounds. Therefore, this novel hemostatic powder has demonstrated both physical and physiological mechanisms for the promotion of coagulation.

**Figure 9:**
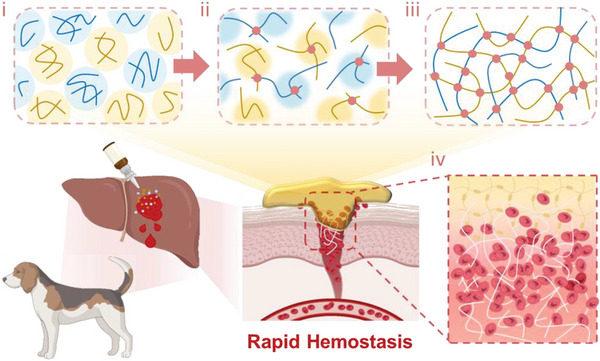
Schematic representation of the hemostatic process of the rapid self‐gelling powder, which has consisted of four stages: i) interfacial water resorption; ii) powder‐hydrogel transformation; iii) tissue wet adhesion and sealing; iv) pro‐coagulant activity.

As evidenced by multiple experiments, this powder has shown to improve the control of hemorrhage in emergency and clinical settings, and the hemostatic process could be divided into four stages (**Figure** **9**): i) the powder will recognize complex morphology, and the superfluous blood on the bleeding wound was quickly absorbed by the hydrophilic nature and great specific surface area; ii) based on the characteristic cross‐linking strategy, the powder has undergone gelling transformation and sequential‐cross‐linking, forming a tight barrier on the wound; iii) the physical entanglement and covalent/non‐covalent interaction with the tissue caused by the dissolved polymer chain and a variety of reactive groups in the modified material can result in excellent wet adhesion; iv) this powder could pro‐coagulate both physically and physiologically, specifically coagulation concentration and fibrinogen capture. Moreover, the gelling transformation and the pro‐coagulation of powder could create a favorable microenvironment for tissue healing, expected to achieve the hemostasis‐tissue repairment cascade.

## 3 Conclusion

In the present study, a hemostatic powder comprised of modified SA and CMCS was prepared with a convenient liquid nitrogen‐assisted grinding method, with an aim to effectively control the non‐compaction hemorrhage. Compared with conventional hemostatic powders, this powder has an innovative material form, i.e., rapid gelling transformation following absorbing plenty of fluid owing to its hydrophilic nature and characteristic cross‐linking strategy (Schiff base and sulfhydryl‐Michael addition reaction). The hydrogel‐transformed characteristics and ingenious chemical design were also beneficial to the formation of a tight physical barrier, where the multiple cross‐linking networks have enhanced the cohesion and fracture resistance of the gel, endowing the powder with excellent tissue wet adhesion and sealing capability. As a prerequisite, the biocompatibility of the material was primarily verified at cellular, blood and tissue levels. In in vivo hemostatic application, the powder, in comparison to CP, showed a significant reduction in blood loss and shortened the bleeding time in models of massive hemorrhage in rats, rabbits and canines, showing an excellent hemostatic effect. Exploration of the coagulation mechanism of the hemostatic powder showed that it could promote rapid hemostasis by the means of physically blocking and inducing the rapid formation of fibrin network. Conceivably, the composite hemostatic powder can effectively combines the powder characteristics and the advantages of hydrogels, which may provide inspiration for the design of novel hemostatic materials and functional dressings. Our hemostatic powder has provided a promising candidate for the treatment of non‐compressible massive hemorrhage.

## 4 Experimental Section

### Synthesis of Modified SA and CMCS

The ADA‐Mal was synthesized through a two‐step modification. The aldehyde group was primarily introduced into the SA through oxidation of NaIO_4_. The oxidized SA (ADA) was obtained by dialyzing for 5 days (MWCO: 3500 Da) and freeze‐drying for 24 h. The maleimide group was grafted into the ADA by amidation reaction for 24 h using Mal‐HCl, EDC, and NHS followed by dialyzing and freeze‐drying. The synthesis method of SA‐Mal was the same as that for ADA‐Mal, except that the raw material was SA instead of ADA. The CMCS was reacted with CAS‐HCl through an amidation reaction for 24 h and dialyzed for 5 days in 0.01 m Na_2_B_4_O_7_ solution (MWCO: 3500 Da). Subsequently, DTT was added to avoid the formation of disulfide bonds before freeze‐drying.

The chemical structure of ADA‐Mal and CMCS‐SH was evaluated by ^1^H NMR (dissolved in D_2_O, 600 MHz, AV II‐400 MHz, Bruker, Switzerland) and FTIR (INVENIO R, Bruker, Switzerland). The degree of oxidation in the ADA was determined with a hydroxylamine hydrochloride method. The substitute ratio of maleimide group and sulfhydryl in ADA‐Mal and CMCS‐SH was characterized by ^1^H NMR, respectively. The details are provided in the Supporting Information.

### Preparation of the AxCy Powders and Related Hydrogels

The A*x*C*y* hemostatic powders (abbr. A*x*C*y*, *x*, *y* = 3, 5, 7) were prepared through convenient grinding. Briefly, freeze‐dried ADA‐Mal, and CMCS‐SH were ground 3 times by high‐energy ball milling (30 Hz, 10 min) following freezing with liquid nitrogen for 15 min. The morphology and particle size of the ADA‐Mal and CMCS‐SH powders were characterized with a scanning electron microscope (SEM, EVO MA10, ZEISS, Germany) and laser particle size meter (Mastersizer 2000, Malvern, UK), respectively.

The A7C3, A5C5, and A3C7 powders were prepared by mixing quantitative ADA‐Mal and CMCS‐SH powder with a mass ratio of 7 : 3, 5 : 5, and 3 : 7, respectively. The prepared A*x*C*y* were kept in a dry environment. The prefabricated A*x*C*y* hydrogels were formed by the solution of ADA‐Mal and CMCS‐SH. Briefly, for the preparation of A*x*C*y* hydrogels, the lyophilized ADA‐Mal and CMCS‐SH were dissolved in PBS at *x*% and *y*% concentrations, respectively. Thereafter, they were rapidly mixed with equal volume for the formation of the hydrogel. Similarly, the ADA‐Mal + CS, SA‐Mal + CS and ADA + CS hydrogels were formed using 3% CMCS‐SH solution in PBS and differently modified SA solution (7% ADA‐Mal, SA‐Mal, and ADA in PBS, respectively) with equal volume. The gelling time of the hydrogel was determined with a tube inversion method. Briefly, 500 µL of 3% CMCS‐SH solution was vortexed, and an equal volume of 7% modified SA solution was added. The tube was then inverted to observe the mixing state, and the gelling time was recorded when the mix occurred no drop.

### Water/Blood Absorption Behavior and Rapid Gelling Capacity of the AxCy

Anticoagulated whole blood was prepared by mixing fresh rabbit blood with 3.8% sodium citrate (1 : 9, v/v). The difference in liquid absorption capacity between the A7C3 powder and prefabricated hydrogel of the same mass was visually assessed, and the remaining dyed PBS was recorded. The water/blood absorption curves within 15 min of the A7C3 powder and prefabricated hydrogel were recorded. The details are provided in the Supporting Information.

The rapid gelation ability of the powder was initially evaluated with an inverted tube method. The pre‐weighed A7C3 powder was poured into the tube containing PBS/blood (1 mL). After 5 s, the tube was inverted and the liquid flow was recorded. The Yunnan BaiYao medicinal powder, a commercially made hemostatic powder (abbr. CP), was used as the control. To better record the process of gelation, the morphology of samples was observed using SEM following rapidly frozen with liquid nitrogen after adding PBS for various time (5, 30, and 300 s). The chemical structure of self‐gelling hydrogel was evaluated by the spectra for C 1s, N 1s, O 1s, and B 1s in the X‐ray photoelectron spectroscopy (XPS, Axis Ultra DLD, Kratos, UK). Following calibrating hydrocarbon peak of C 1s (284.6 eV), the results was fitted using CasaXPS software.

### Mechanical and Rheological Properties of the AxCy

To ensure the reliability of the experiments for the evaluation of mechanical and rheological properties, the self‐gelling process of the A7C3, A5C5, and A3C7 powder was adjusted so that the total concentration of ADA‐Mal and CMCS‐SH in the self‐gelling hydrogel was 10%. The compressive strength (KPa) was analyzed with uniaxial compression test using an universal mechanical testing machine (Instron 5967, USA) (*n* = 3). The swelling and degradation properties were used to evaluate the stability of the A7C3, A5C5, and A3C7 powder in vitro. The details are provided in the Supporting Information.

The rheological properties were assessed using a rotational rheometer (Physica MCR302, Anton Paar, Austria). The storage modulus (G’) and loss modulus (G’’) were recorded at a fixed angular frequency, strain and temperature (1 Hz, 1% and 37 °C). The alternate step strain sweep with the oscillatory strain switched back and forth between 1% and 300% for each interval was carried out to characterize the self‐healing properties of the samples. Meanwhile, to assess the macroscopic shape adaptivity behavior, the self‐gelling A7C3 hydrogel was placed in “S”, “C”, and “U”‐shaped molds, which were then removed. The details are provided in the Supporting Information.

### Tissue Wet Adhesive and Sealing of the AxCy

The adhesive strength (KPa) of the A7C3, A5C5, and A3C7 powders were evaluated with a lap‐shear test on an universal mechanical testing machine (Instron 5967, USA) according to modified ASTM F2255‐05. To estimate the capability for wound closure, a modified bursting test was used to determine the burst pressure (KPa), with a commercially made fibrin glue (Guangzhou Bioseal Biotech Co., Ltd., China) used as the control. Subsequently, a series of macroscopic adhesion experiments using pig skin, small intestine and stomach were carried out. The details are provided in the Supporting Information.

### Biocompatibility of the A_x_C_y_

The cyto‐, hemo‐ and histo‐compatibility of the A7C3, A5C5, and A3C7 powders were evaluated with cell proliferation (CCK‐8 assay, Cat No. 40203ES60; Yeasen, Shanghai, China), cell viability (Live/Dead staining), hemolysis assay, and subcutaneous implantation, respectively. The NIH‐3T3 and HSF cells were cultured in Dulbecco's modified eagle medium (DMEM) and DMEM/F‐12 (1 : 1, v/v) containing 10% fetal bovine serum and 1% penicillin‐streptomycin, respectively. The systemic toxicity of the materials was evaluated by blood biochemistry, blood routine and HE staining of major organs. The in vivo biodegradation of A7C3 powder was evaluated by subcutaneous implantation in rats. All animal experiments were approved by Sichuan University Animal Care and Use Committee and carried out in accordance with relevant principles (No. 20 220 225 127). Relevant details are provided in the Supporting Information.

### In Vitro Blood‐Related Component Preparation

As mentioned above, anticoagulated whole blood was prepared by mixing fresh rabbit heart blood with 3.8% sodium citrate (1: 9, v/v). The platelet rich plasma (PRP) was prepared by centrifugation of anticoagulated whole blood at 1000 rpm for 10 min. Thereafter, the erythrocytes were carefully collected from the erythrocyte layer and washed with PBS for 3 times (1000 rpm, 10 min).

### In Vitro Pro‐Coagulant Properties of the AxCy

The pro‐coagulant property of the A7C3, A5C5, and A3C7 powders was first evaluated with a blood clotting time test. Briefly, 10 mg of the material was added into 50 µL of working solution (anticoagulated whole blood : 0.1 m CaCl_2_ solution = 9 : 1) in a 96‐well plate, and the well was gently washed with PBS for the predetermined time. The time that the clot forms uniformly and stably after washing was defined as the blood clotting time. A blood clotting index (BCI) assay was used to measure the tendency to pro‐coagulation. The adherent platelets on the A7C3, gauze and fibrin glue were quantified with a lactate dehydrogenase (LDH) assay kit (Nanjing Jiancheng Bioengineering Institute, China). After the treatment, free fibrinogen (FIB) in the blood was detected with an automatic coagulometer, and the morphology of the platelets and erythrocytes on the A7C3, gauze and fibrin glue was observed by SEM treated by critical point drying and gold sputtering. The details are provided in the Supporting Information.

### In Vivo Hemostasis Properties of the AxCy Powders

The in vivo hemostatic performance of the A7C3 and CP was first assessed with a rat bleeding liver model. Male SD rats (weighing 300–350 g each) were placed in supine position and anesthetized, and an abdominal incision was made on the belly after shaving. After cleaning up excess liquid with gauze, the liver was carefully dissected and put on a pre‐weighed filter paper. Half of liver was cut with a scalpel, and the bleeding time was recorded. Thereafter, 100 mg of the A7C3 and CP was applied for the hemostasis. Rats without treatment were used as the control group. The blood loss was calculated by weighing the blood on the filter paper (*n* = 3). The bleeding site was further observed by SEM after fixing, critical point drying and spraying with gold.

The hemostatic capability was further assessed with a rabbit liver defect and central auricular artery bleeding model. Male New Zealand white rabbits (weighing 2.5–3.0 kg each) were anesthetized with pentobarbital sodium (1.1 mL kg^−1^). To construct the non‐compressible liver injury hemorrhage model, a deep wound (1 cm in diameter and depth) was created by a skin biopsy trephine in the rabbit liver, and 300 mg of the materials (A7C3 and CP) was immediately applied for hemostasis. Moreover, the needle tip (22G) was used to create rabbit central auricular artery bleeding, and the materials were positioned on the bleeding site followed by using weight (100 g) to press the proximal part for 30 s. The bleeding process was then photographed.

To further evaluate the hemostatic effect in large animals, a canine liver defect non‐compressible hemorrhage model was constructed. Beagle dogs weighing 10–15 kg each were anesthetized with pentobarbital sodium (1 mL kg^−1^). A rotary skin biopsy trephine was used to create a deep wound with a diameter and depth of 1.5 cm in the liver. Five hundred milligrams of the materials (A7C3 and CP) were rapidly applied. The bleeding time and blood loss were recorded with the aforementioned method (*n* = 3). One week after the treatment, the dogs were euthanized by over‐dose pentobarbital sodium, and the livers were collected for the H&E staining.

### Statistical Analysis

All data were presented as mean ± standard deviation. Statistical significance between the groups was assessed by one‐way ANOVA and Student's t‐test. *P* < 0.05 was considered to be statistically significant (*P < 0.05, **P < 0.01, ***P < 0.001, ****P < 0.0001).

## Conflict of Interest

The authors declare no conflict of interest.

## Supporting information

Supporting Information

Supplemental Movie 1

Supplemental Movie 2

Supplemental Movie 3

## Data Availability

The data that support the findings of this study are available from the corresponding author upon reasonable request.
