# Supplementary material for: A Self‐Assembly Pro‐Coagulant Powder Capable of Rapid Gelling Transformation and Wet Adhesion for the Efficient Control of Non‐Compressible Hemorrhage
Source: Adv Sci (Weinh). 2023 Dec 3;11(4):2306289. doi: 10.1002/advs.202306289 (PMC10811489; doi:10.1002/advs.202306289)
Supplement: Supplementary file 1 — Supporting Information [file ADVS-11-2306289-s004.pdf]

ADVANCED SCIENCE
Open Access

Supporting Information

for *Adv. Sci.*, DOI 10.1002/advs.202306289

A Self-Assembly Pro-Coagulant Powder Capable of Rapid Gelling Transformation and Wet Adhesion for the Efficient Control of Non-Compressible Hemorrhage

*Xiong-Xin Lei, Chen-Yu Zou, Juan-Juan Hu, Ming-Hui Fan, Yan-Lin Jiang, Ming Xiong, Chen Han, Xiu-Zhen Zhang, Ya-Xing Li, Long-Mei Zhao, Rong Nie, Jesse Li-Ling and Hui-Qi Xie**

Supporting Information

A Self-assembly Pro-Coagulant Powder Capable of Rapid Gelling Transformation and Wet Adhesion for the Efficient Control of Non-Compressible Hemorrhage

*Xiong-Xin Lei, Chen-Yu Zou, Juan-Juan Hu, Ming-Hui Fan, Yan-Lin Jiang, Ming Xiong, Chen Han, Xiu-Zhen Zhang, Ya-Xing Li, Long-Mei Zhao, Rong Nie, Jesse Li-Ling, and Hui-Qi Xie**

Xiong-Xin Lei, Chen-Yu Zou, Ming-Hui Fan, Yan-Lin Jiang, Chen Han, Xiu-Zhen Zhang, Ya-Xing Li, Long-Mei Zhao, Rong Nie, Jesse Li-Ling, Hui-Qi Xie

Department of Orthopedic Surgery and Orthopedic Research Institute, Laboratory of Stem Cell and Tissue Engineering, State Key Laboratory of Biotherapy, West China Hospital, Sichuan University, Chengdu, Sichuan 610041, P. R. China

E-mail: xiehuiqi@scu.edu.cn

Chen-Yu Zou, Ming-Hui Fan, Yan-Lin Jiang, Chen Han, Xiu-Zhen Zhang, Ya-Xing Li, Long-Mei Zhao, Rong Nie, Jesse Li-Ling, Hui-Qi Xie

Frontier Medical Center, Tianfu Jincheng Laboratory, Chengdu, Sichuan 610212, P. R. China.

Juan-Juan Hu, Ming Xiong

Department of Otolaryngology, Head and Neck Surgery, West China Hospital, Sichuan

University, Chengdu, Sichuan 610041, P. R. China

Jesse Li-Ling

Center of Medical Genetics, West China Second University Hospital, Sichuan University, Chengdu, Sichuan 610041, P. R. China

Xiong-Xin Lei

Department of Orthopedic Surgery, First People's Hospital of Foshan, Foshan, Guangdong 528000, P. R. China

Experimental section:

***Materials*:**

Sodium alginate (SA, 200 ± 20 mps), sodium periodate ($NaIO_4$, 98%), sodium tetraborate decahydrate ($Na_2B_4O_7 \cdot 10H_2O$, 99.5%), L-cysteine hydrochloride (CSA-HCl, $C_3H_7NO_2S \cdot HCI$, 98%), cysteine hydrochloride (Mal-HCl, $C_2H_7NS \cdot HCl$, 98%), dithiothreitol (DTT, $C_4H_{10}O_2S_2$, 99%), ethylene glycol ($C_2H_6O_2$, 99.8%), N-(3-dimethylaminopropyl)-N′-ethylcarbodiimide hydrochloride (EDC, 98%) and N-hydroxysuccinimide (NHS, 98%) were purchased from Shanghai Aladdin Bio-Chem Technology Co., Ltd. Carboxymethyl chitosan (CMCS, degree of substitution: 90%) and 2-(N-morpholino) ethanesulfonic acid monohydrate (MES, $C_6H_{13}NO_4S \cdot H_2O$) were purchased from Beijing Solarbio Science & Technology Co., Ltd. All reagents were used without further purification.

***Synthesis of modified SA and CMCS*:**

The ADA-Mal was synthesized through a two-step modification. Briefly, 10 g of SA was dispersed in 50 mL of ethanol, to which $NaIO_4$ aqueous solution (2.5 g, 5% w/v) was added dropwise. The reaction was lasted for 6 h in the darkness and terminated by the addition of 15 mL of ethylene glycol for 30 min. The product (oxidized SA, ADA) was obtained after dialyzing for 5 days (MWCO: 3500 Da) and freeze-drying for 24 h. For chemical grafting, ADA (2 g, about 10 mmoL) dissolved in MES buffer (pH 5.5) at a concentration of 1% w/v was reacted with 1.7 g Mal-HCl (15 mmoL) with the catalysis of EDC/NHS (15 mmoL, 1.725 g and 2.87 g, respectively). The reaction was lasted for 24 h, and the ADA-Mal was obtained following the dialysis and freeze-drying as described above.

Sulfhydrated CMCS was prepared through the amidation reaction. Briefly, 4 g of CMCS (about 20 mmoL) was completely dissolved in deionized water (200 mL). Thereafter, EDC (5.74 g, 30 mmoL) and CSA-HCl (3.4 g, 30 mmoL) were added, and the reaction has lasted for 24 h. Upon completion, CMCS-SH was harvested and dialyzed for 5 days in 0.01 M $Na_2B_4O_7$ solution (MWCO: 3500 Da) followed by addition of 2 g of DTT before freeze-drying. The freeze-dried CMCS-SH was added into 500 mL ethanol and shook in 4 °C for 1 h to clear the superfluous DTT, and the ethanol was then removed by vacuum filtration.

***Water/blood Absorption Behavior and Rapid Gelling Capacity of the AxCy*:**

To compare the water absorption behavior of the hydrogel and the powder, 300 μL of dyed PBS was absorbed within 3 min by the A7C3 hydrogel and powder of the same weight, and the residual PBS was recorded. The liquid absorption of the A7C3 hydrogel and powder were

further quantified (n = 3). Briefly, pre-weighed hydrogel and powder were placed in a glass container, to which the water/blood was rapidly added. Thereafter, at the set intervals (0.5, 1, 3, 5, 10, 15 min), they were weighed after gently blotting the excessive water from the surface after removing the excess liquid. The liquid absorption was calculated as follows:

$$Liquid\ Absorption\ (\%) = \frac{W_t - W_0}{W_0} \times 100\% \tag{1}$$

Where W_0 and W_t respectively referred to the weight of the samples at the initial and preset time.

***Mechanical and Rheological Properties of the AxCy*:**

For uniaxial compression test, cylindrical hydrogel (8 mm in both diameter and height) was prepared. The stress-strain curves of the A7C3, A5C5 and A3C7 were derived with a universal mechanical testing machine with 500 N load cell at a crosshead speed of 5 mm min^{-1}, and the compressive strength (KPa) was calculated by the following formula:

$$Compressive\ strength\ (KPa) = \frac{F\ (N)}{A\ (m^2)} \times 10^3 \tag{2}$$

Where *F* and *A* referred to the force and area of thrust surface on the sample, respectively. The suddenly decreasing stress was considered the indicator of destruction of the sample and recorded as compressive strength.

A rotational rheometer was used to assess the rheological properties of the hydrogels. All experiments were carried out with a cone plate rotor (CP25-3, diameter: 25 mm, angle: 3°). The time sweep mode (1%, 1 Hz, 37 °C) was used to evaluate the storage modulus (G') and loss modulus (G''). The self-healing property of the A7C3, A5C5 and A3C7 hydrogels was determined under the alternate step strain sweep mode, which fixed the angular frequency and

temperature at 1 Hz and 37 °C, the oscillatory strain has ranged between 1% and 300% for each interval. Meanwhile, the shape adaptivity of the A7C3 hydrogel was evaluated by using "S", "C" and "U"-shaped molds. Briefly, the self-gelling hydrogel was placed into the blank molds which were removed after 5 min, leaving the hydrogel with the special shapes.

***Swelling and degradation behavior of the AxCy*:**

For the swelling test, pre-weighed A7C3, A5C5 and A3C7 were placed in a glass vial filled with PBS at 37°C and 60 rpm (n = 3) and, at set interval, weighed after gently blotting the extra water. The swelling ratio (%) was calculated with the following formula:

$$\text{Swelling Ratio (\%)} = \frac{W_{\mathrm{t}} - W_0}{W_0} \times 100\%$$

Where W_0 and W_t referred to the weight of the hydrogel at the initial and pre-set time, respectively. The degradation test of the A7C3, A5C5 and A3C7 was carried out in a similar way. Briefly, the weighed hydrogels placed in the vial filled with PBS were incubated at 37°C and the samples were freeze-dried and weighed per 3 days. The weight remaining ratio (%) of samples was calculated with the following formula:

$$\text{Remain Mass (\%)} = \frac{W_{\mathrm{t}} - W_0}{W_0} \times 100\%$$

Here, the W_0 and W_t have referred the weight of the original and remaining hydrogels after various periods of degradation, respectively.

***Wet Adhesion of the AxCy*:**

For lap-shear test, two PMMA slices (30 × 10 × 2 mm^3) with hog casing (10 × 10 mm^2) were soaked in PBS (37 °C, 30 min) to simulate the wet tissue. The A7C3, A5C5 and A3C7 hydrogels (100 mg each) were then placed into the wet hog casing. After stabilizing for 30

min, the lap-shear test was conducted (1 mm min^{-1}), and the adhesion strength (KPa) of the hydrogels was analyzed with force-displacement curves (n = 3). A commercially made fibrin glue (Guangzhou Bioseal Biotech Co., Ltd., China) was applied by following the manufacturer's instructions. The wet adhesive ability of the A7C3 was evaluated with a pigskin wet-adhesion experiment. PBS was added to a clean pigskin, which was then covered with a glass sheet with the A7C3 (fixed by glue). After 10 s, the glass sheet was lifted. For the two-pigskin adhesion experiment, two clean pigskins were immersed in the PBS, and the A7C3 was poured onto them, followed by overlapping and lifting. A water scour experiment was used to assess the adhesion stability of the powders. The A7C3 was gelled with the pigskin and stabilized for 10 min before rushed by water. Simultaneously, the morphology of the adhesive surface between the material and the tissue was observed by SEM after the lyophilization.

***Tissue Sealing of the AxCy*:**

To evaluate the wound sealing ability, a modified bursting test was carried out to determine the burst pressure (n = 3). The tissue with a hole 2 mm in diameter was secured to a closed device by T-junction, which was also connected to a pressure gauge and an injection pump. The hole was closed by the A7C3, A5C5 and A3C7, and the injection pump had run at a speed of 5 mL min^{-1} following the hydrogel stabilization for 5 minutes, whilst the pressure on the gauge was recorded. The peak pressure was deemed as the burst pressure (n = 3). Thereafter, a series of wound sealing experiments were carried out on the small intestine and stomach samples. A needle tip (12G) was used to create a hole in the small intestine and stomach. The

moderate powder has sealed the wound following addition of water, and the effect of wound closure was assessed.

***Biocompatibility of the AxCy*:**

To evaluate the cytocompatibility, extracts of the A7C3, A5C5 and A3C7 were prepared in complete medium (30 mg mL^{-1}) at 37 °C for 24 h. The NIH-3T3 cells (1500 cells $well^{-1}$) and HSF cells (1500 cells $well^{-1}$) were placed in 96-well plates, respectively, and the extract (100 μL) was replaced after the cells had attached. Thereafter, a CCK-8 assay was conducted by following the manufacturer's instruction to estimate the cell proliferation at 1, 3 and 5 days with complete medium as the control (n = 3). To determine the survival and status, the cells were cultured in the extract for 3 days. Using a Live/Dead cell double staining kit (Dojindo, Japan), the living and dead cells were respectively stained by calcein acetoxymethyl ester (Calcein-AM) and PI (1 μL mL^{-1}) at 37 °C for 10 min and observed under an inverted fluorescence microscope.

The hemocompatibility of the hydrogels was measured with a hemolysis test. Briefly, the erythrocytes suspension (5% v/v in PBS) was added after 500 μL of the A7C3, A5C5 and A3C7 hydrogels were evenly placed on the bottom of a 48-well plate (n = 3). After 1 h of incubation, the suspension was collected and centrifuged at 1000 rpm for 10 min. The absorbance value at 540 nm was measured with a microplate reader. PBS and Triton X-100 (0.1%) were respectively used as the negative and positive controls. The hemolysis ratio was calculated with the following formula:

$$\textit{Hemolysis Ratio (\%)} = \frac{A_S - A_{NC}}{A_{PC} - A_{NC}} \times 100\% \tag{3}$$

Where the A_S, A_{PC} and A_{NC} represented the absorbance values (540 nm) of the sample, Triton X-100 and PBS, respectively.

To assess the histocompatibility, the samples were implanted subcutaneously into the SD rats, which were randomly divided into three groups (A7C3, A5C5 and A3C7) (n = 3). Prior to the operation, the pre-weighed disc-shaped samples were shaped in a silicone mold (10 mm in diameter) and sterilized with UV irradiation for 6 h. The rats were then anesthetized with chloral hydrate, and their backs were shaved. A 2-cm-long incision was made on the back, and the hydrogel samples were carefully implanted subcutaneously. The rats were euthanized at preset time. The implant site, heart, liver, spleen, lung and kidney of the rats were sampled for H&E staining after fixation with 4% paraformaldehyde, embedding and sectioning. The organs from healthy rats were used as the controls. Meanwhile, arterial blood was collected for routine blood and biochemical assays (Sysmex CS-5100, Japan) (n = 3). To further assess the biodegradation of materials, the sterile A7C3 hydrogel prepared with the previously described method was implanted subcutaneously in rats. On days 7, 14 and 21, the rats were anesthetized with chloral hydrate. The implants were tested using ultrasonography (Mindray, M9vet), and the implants thickness (cm) were measured using the software that comes with the instrument (n = 3). After euthanasia, the implants and surrounding tissue were photographed.

***In Vitro Pro-coagulant Properties of the AxCy*:**

A blood clotting index (BCI) assay was carried out with a previously described method with modification. In brief, anticoagulant whole blood (10 μL) was evenly dripped onto the surface

of the pre-heated A7C3, A5C5 and A3C7 (n = 3). The materials were incubated at 37 °C for 1, 3 and 5 min after adding $CaCl_2$ solution (1 μL, 0.2 M). The dissociative erythrocytes were then lyzed by slow addition of 2 mL of H_2O after the incubation (37 °C), and the absorbance of supernatant at 540 nm was measured with a microplate reader. A blank control was set by the addition of H_2O (2 mL) to 10 μL blood. The BCI was calculated with the following formula:

$$BCI\ (\%) = \frac{A_S}{A_B} \times 100\% \tag{4}$$

Where A_S and A_B were the absorbance values of the sample and the blank control, respectively.

A LDH assay kit was used to quantify the number of platelets adhered to the surface of fibrin glue, gauze and A7C3. Firstly, the platelets was counted under a microscope after 500 times dilution. By following the manufacturer's instruction, the standard curve of platelet count and absorbance at 450 nm was established. 200 μL of PRP was added to the materials (n = 3) in a 24-well plate. After incubating for 60 min, the supernatant was removed, and free platelets were washed away with PBS. The platelets on the surface of the materials were lysed with 1% Triton X-100 (1 mL) and detected with a LDH assay kit. Ultimately, the number of adherent platelets on the hydrogel surface was determined with a standard curve.

To observe the adherent erythrocytes and platelets, the erythrocyte suspension (50% v/v, 200 μL) and PRP (200 μL) was respectively incubated on the surface of fibrin glue, gauze and A7C3 for 10 min and 60 min. After washing with PBS gently, the samples were fixed with 4% paraformaldehyde and dehydrated through a ethanol gradient. The morphology of platelets

and erythrocytes on the sample surface was examined by SEM after critical point drying and gold sputtering treatment.

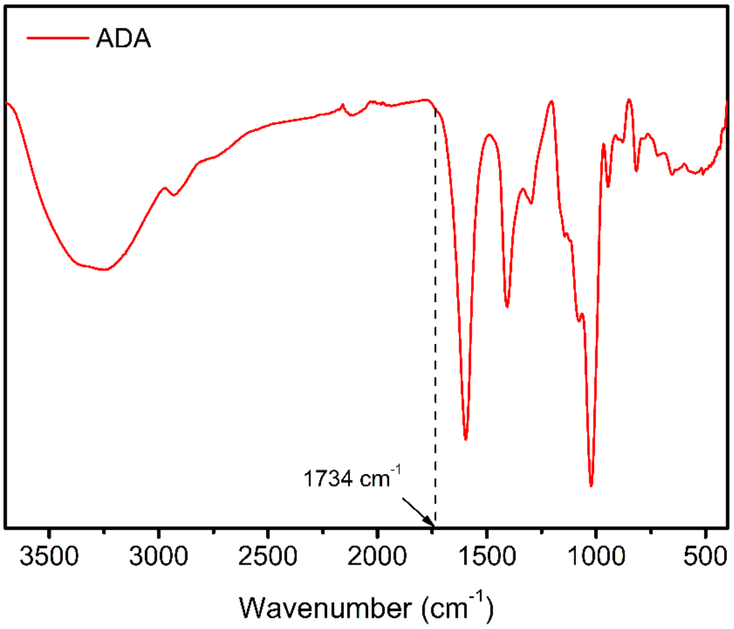

Figure S1. The FTIR spectra of oxidized sodium alginate (ADA)

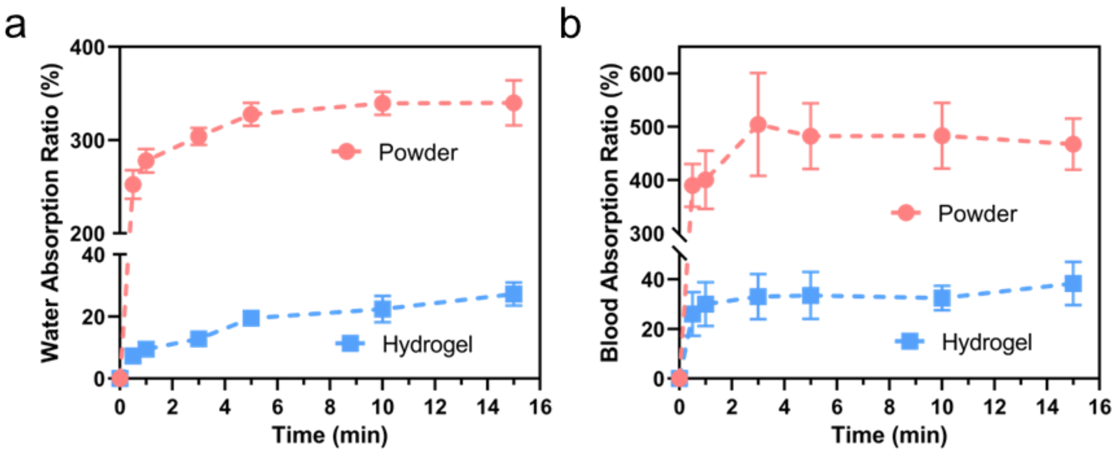

Figure S2. The absorption ratio of the A7C3 powder and hydrogel in a) water and b) blood

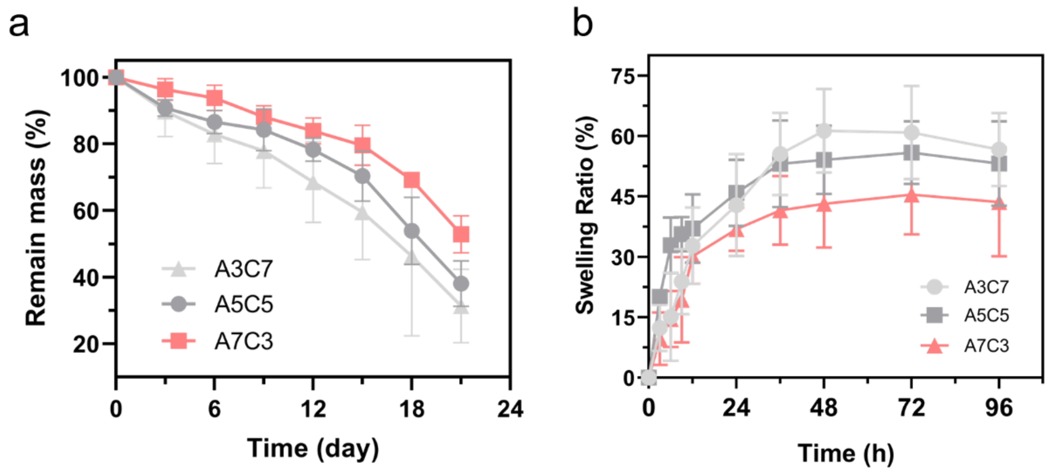

Figure S3. The a) degradation curves and b) swelling curves of the A7C3, A5C5 and A3C7

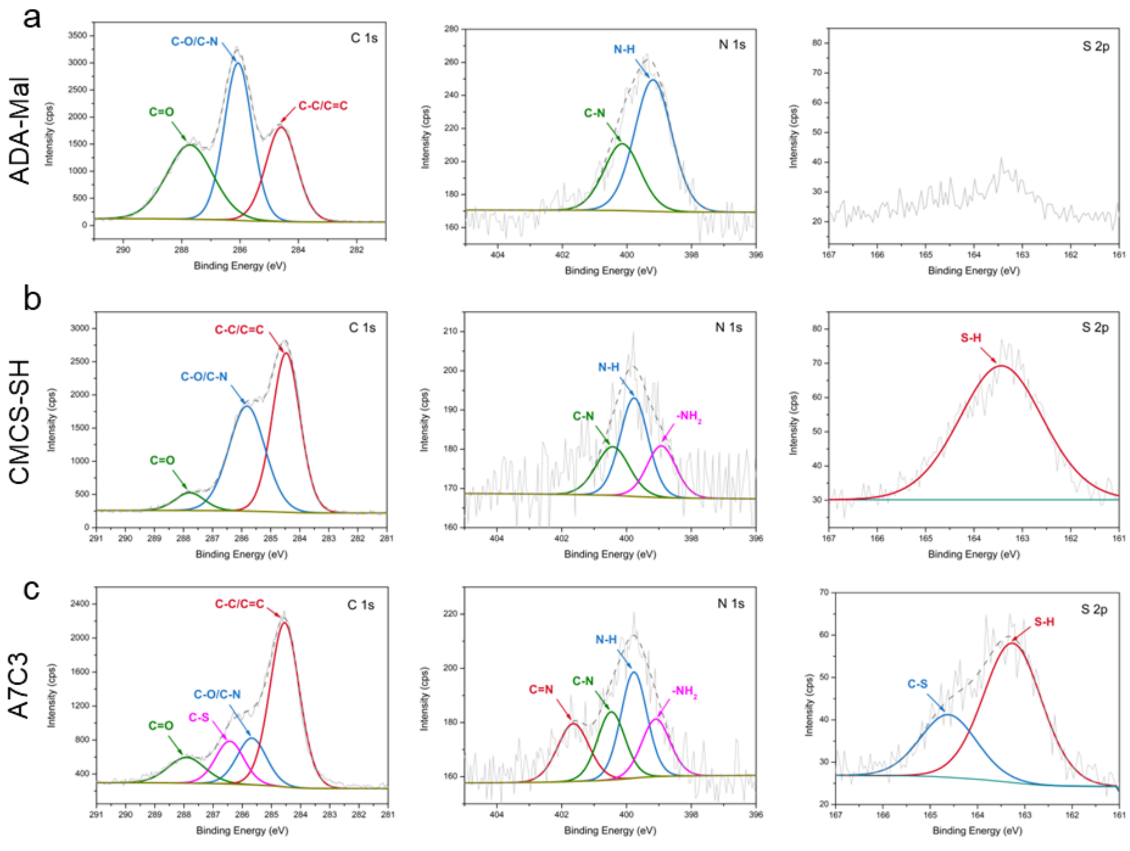

Figure S4. XPS spectra (C 1s, N 1s, S 2p) of a) ADA-Mal, b) CMCS-SH and c) A7C3

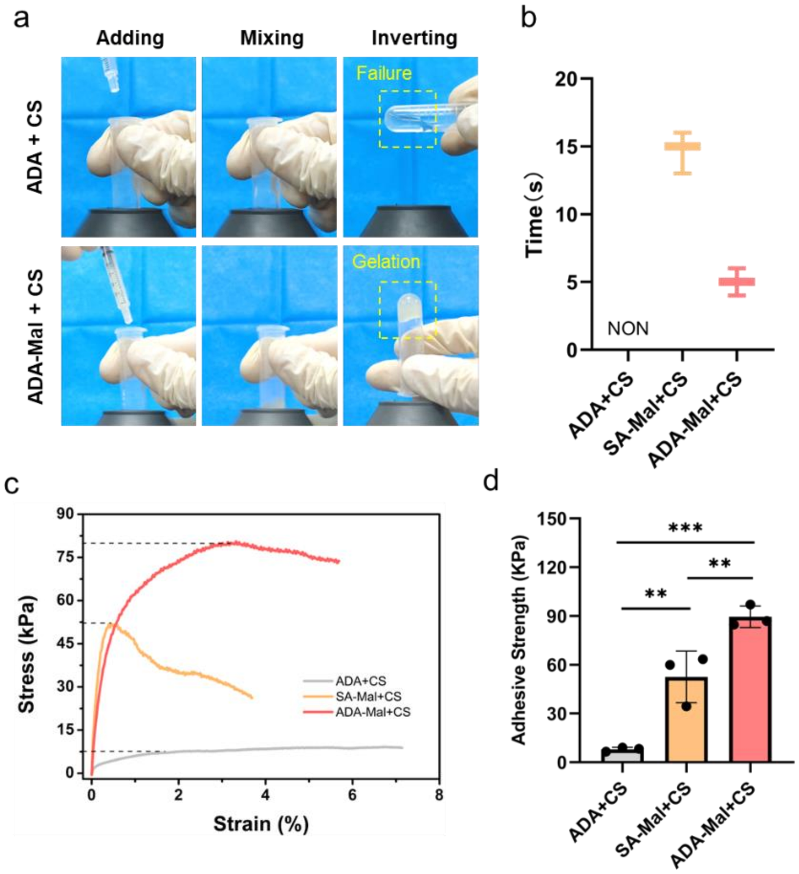

Figure S5. a) The gelation process when the ADA or ADA-Mal was added into the CS; b) gelling time, c) stress-strain curves and d) adhesive strength of the ADA + CS, SA-Mal + CS, and ADA-Mal + CS hydrogels

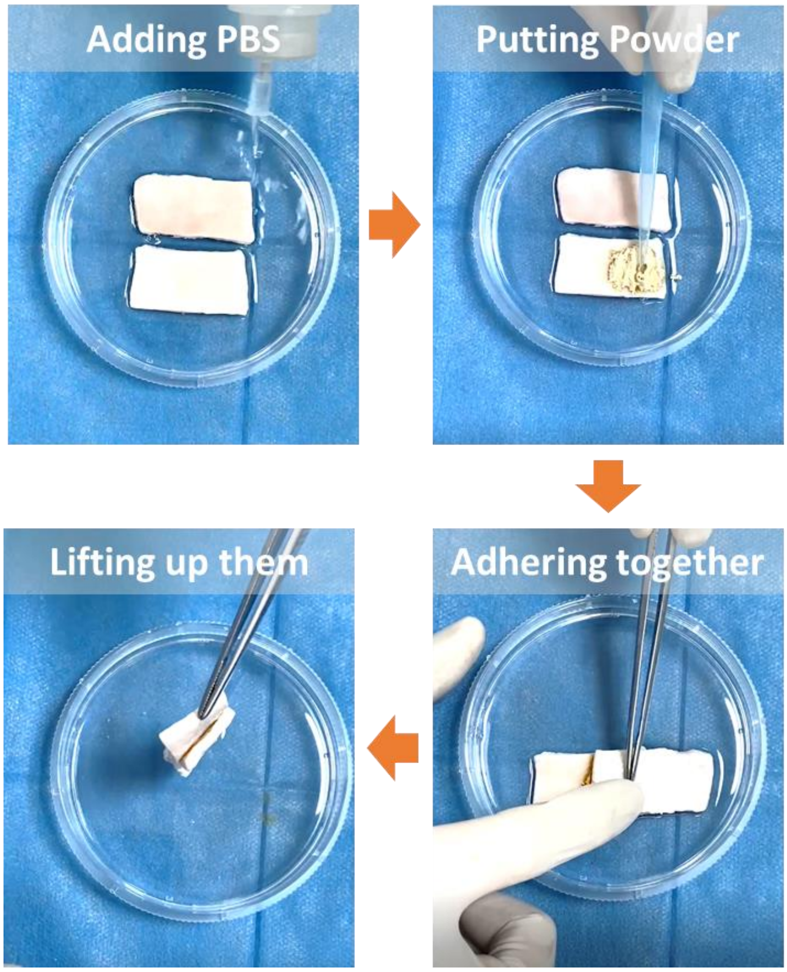

Figure S6. The wet adhesion evaluation of the A7C3

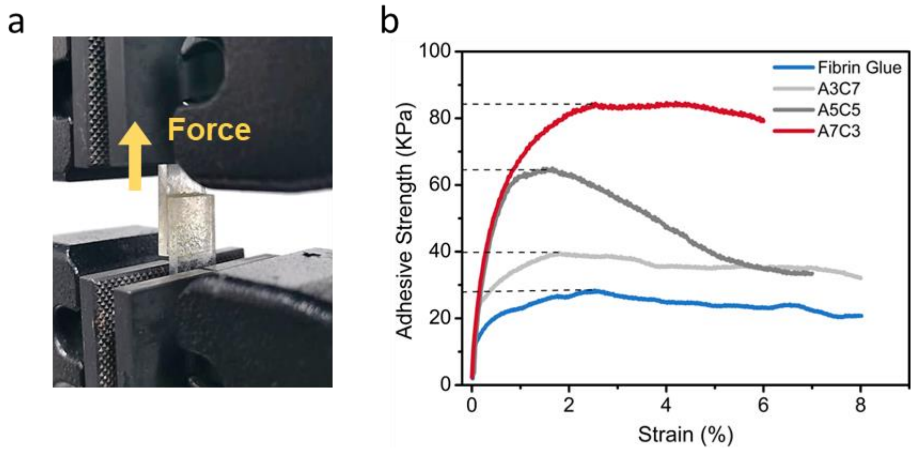

Figure S7. a) Process image and b) stress-strain curves of the A7C3, A5C5, A3C7 and fibrin glue for the lap shear test

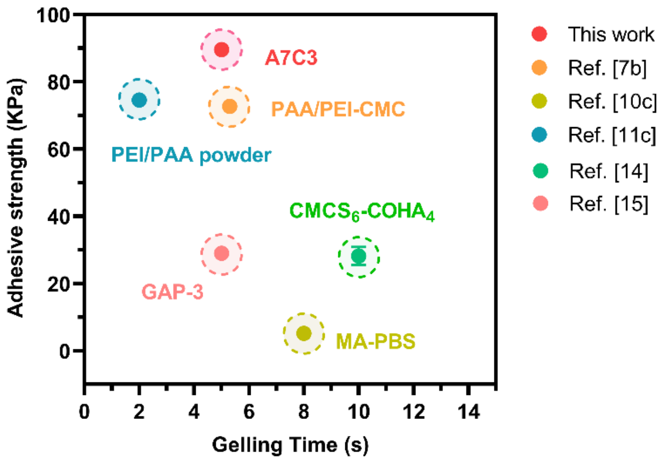

Figure S8. Comparison of self-gelling powder for gelling time and adhesive strength

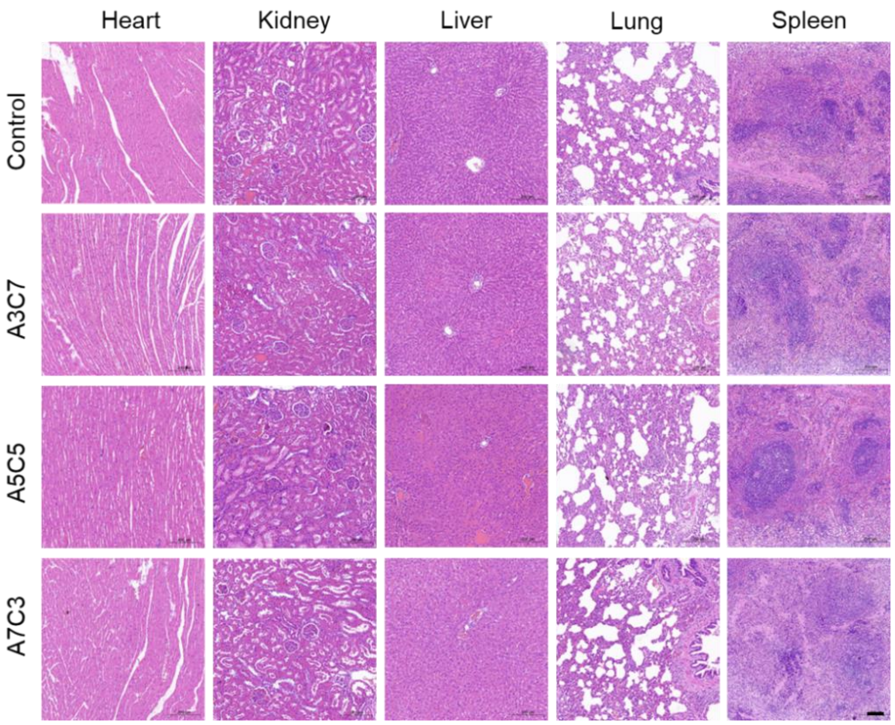

Figure S9. H&E staining of the heart, liver, spleen, lung, and kidney tissues 7 days after the subcutaneous implantation of the A7C3, A5C5 and A3C7 hydrogels, Scale bar = 100 μm

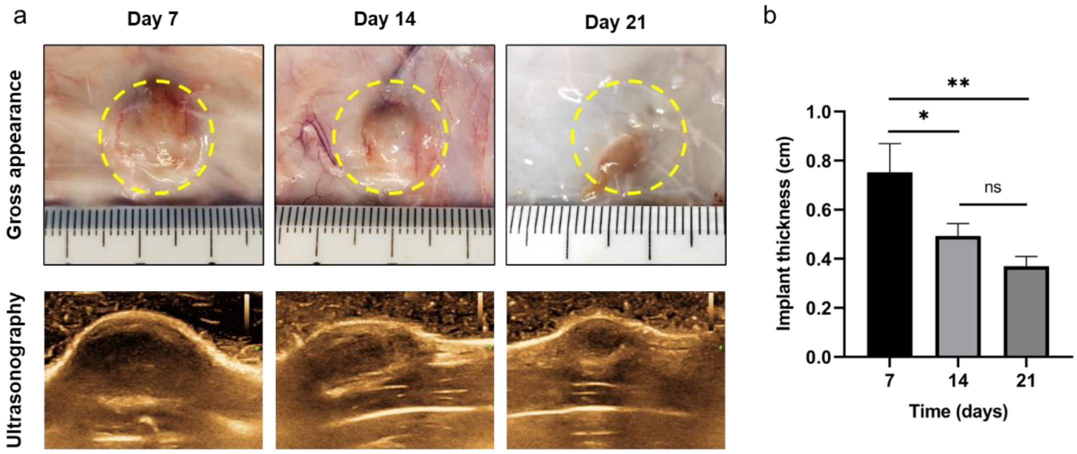

Figure S10. a) Gross appearance, ultrasonography and b) implant thickness of the A7C3 hydrogel after 7, 14 and 21 days of subcutaneous implantation (**P* < 0.05, ***P* < 0.01, ns: no significant difference)

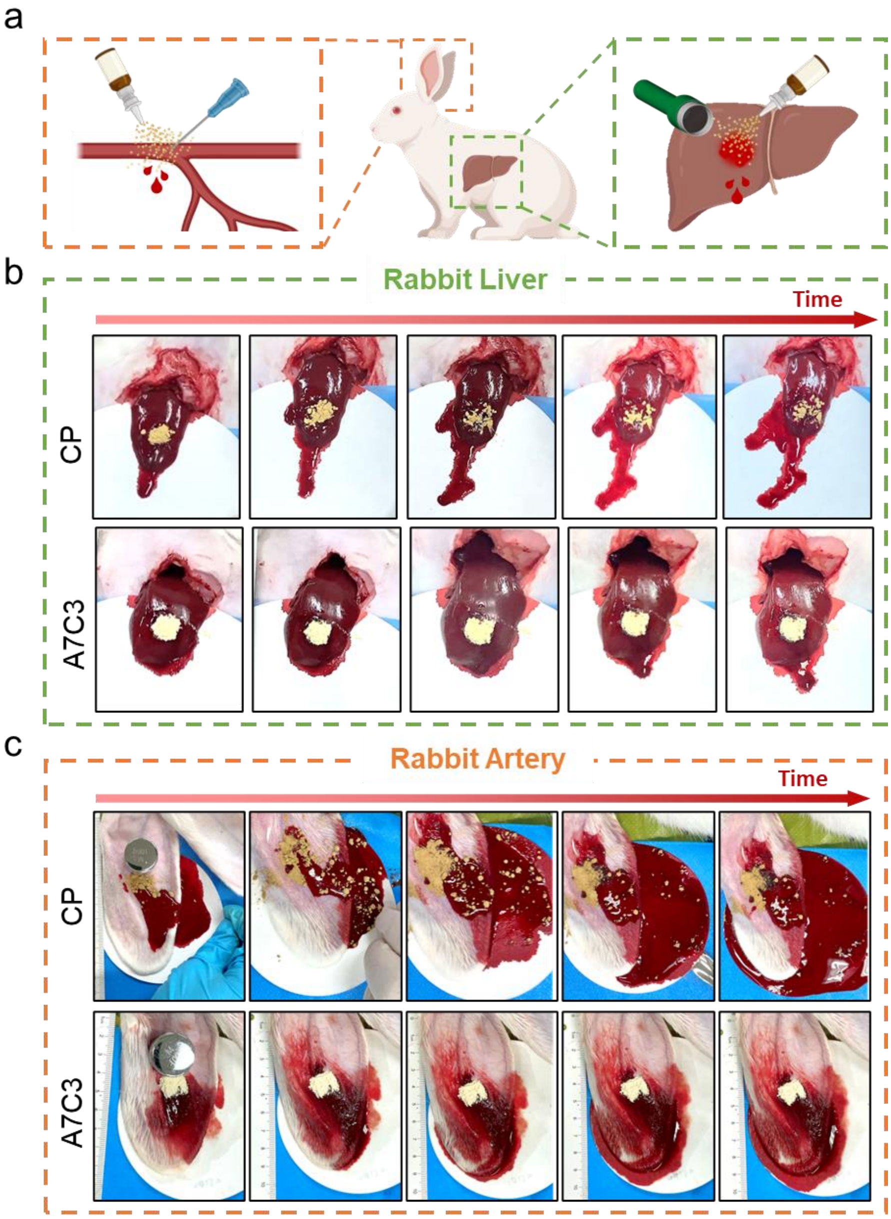

Figure S11. a) Schematic diagram of the non-compressible hemorrhage from the rabbit liver defect and central auricular artery bleeding model. The process of hemostasis using the A7C3 and CP in b) rabbit liver defect non-compressible hemorrhage model and c) rabbit central auricular artery bleeding model
